# Estimation of Soil Surface Roughness Using Stereo Vision Approach

**DOI:** 10.3390/s21134386

**Published:** 2021-06-26

**Authors:** Afshin Azizi, Yousef Abbaspour-Gilandeh, Tarahom Mesri-Gundoshmian, Aitazaz A. Farooque, Hassan Afzaal

**Affiliations:** 1 Department of Biosystems Engineering, Faculty of Agriculture and Natural Resources, University of Mohaghegh Ardabili, Ardabil 56199-11367, Iran; aziziafshin7621@gmail.com (A.A.); mesrigtm@uma.ac.ir (T.M.-G.); 2 Faculty of Sustainable Design Engineering, University of Prince Edward Island, Charlottetown, PE C1A4P3, Canada; hafzaal2@upei.ca; 3 School of Climate Change and Adaptation, University of Prince Edward Island, Charlottetown, PE C1A 4P3, Canada

**Keywords:** stereo vision, soil roughness, tillage, depth map

## Abstract

Soil roughness is one of the most challenging issues in the agricultural domain and plays a crucial role in soil quality. The objective of this research was to develop a computerized method based on stereo vision technique to estimate the roughness formed on the agricultural soils. Additionally, soil till quality was investigated by analyzing the height of plow layers. An image dataset was provided in the real conditions of the field. For determining the soil surface roughness, the elevation of clods obtained from tillage operations was computed using a depth map. This map was obtained by extracting and matching corresponding keypoints as super pixels of images. Regression equations and coefficients of determination between the measured and estimated values indicate that the proposed method has a strong potential for the estimation of soil shallow roughness as an important physical parameter in tillage operations. In addition, peak fitting of tilled layers was applied to the height profile to evaluate the till quality. The results of this suggest that the peak fitting is an effective method of judging tillage quality in the fields.

## 1. Introduction

It takes enormous energy to prepare land for planting in the agriculture sector. In order to record and analyze the quality of tillage operations, it is important to measure and monitor physical parameters, such as soil aggregate distribution and surface roughness [1]. These parameters, which often occur at the soil surface, can be examined more accurately and optimally using recent methods [2,3].

Soil surface roughness is a key parameter which has an important role for understanding and analyzing different soil properties and physical processes which have a direct or indirect relationship with phenomena such as soil erosion, surface water flow movement, agronomy, meteorology, and risk assessment [4].

Soil surface roughness is mainly caused by two factors: rainfall and tillage operations (primary and secondary tillage), particularly when many different tilling machines are used. In other words, tillage operations can increase the roughness of the soil surface, which can in some cases reach decimeters. As a result, soil roughness and soil clods must be considered when evaluating the quality of plowed soil [5].

In practical applications, soil roughness is considered as surface variations that depend on the aggregates in the peaks and depressions on the soil surface. There are several methods to measure this including roller chain, which is a classical method of measuring soil roughness. Although roller chain is accurate and can be used as a reference to evaluate new methods, this method is time consuming and fastidious, and for this reason alternative approaches, especially image-based methods, have been proposed [6,7]. Indeed, irregularities in the soil surface (roughness) are calculated based on different methods, mostly non-contact indicators of surface elevation points measurements [8].

Thanks to the development of new instrumentation and analysis tools, soil surface analysis can now be visualized with image processing [9]. This has been used to create 2D images of soil surface by various different researchers [6,10,11]. With contrast analysis, they obtained good results on average for soil clods diameter estimation but also obtained individual discrepancies. This challenge was solved somewhat by extracting textural features from clods. However, the accuracy was still low [12].

In recent years, several digital elevation models (DEMs) have been used to estimate soil surface roughness [13,14,15,16,17]. DEMs often involve using laser scanners and/or stereo photogrammetry. Because soil clods have geometric features, DEMs provide accurate information about their 3D characteristics. As the pixel values of these kind of images represent altitude, DEM can be used to measure geometrical specifications of clods such as length, width, volume, etc., [18]. Despite the advantages of DEM in studying soil roughness, this method takes a disadvantageously long time in the image acquisition phase which restricts its practical application.

In [19], an automatic photogrammetric method was developed to measure soil surface roughness. Three-dimensional models were calculated from the images taken at millimeter level and were used to extract different roughness indices. The reported results were very satisfactory; however, the procedure used to gather the dataset can be considered as a limitation of this study because the images were taken under the shade of an umbrella, which can affect real-time usage. Additionally, different old and new methods for measuring soil roughness, including contact and non-contact techniques, were introduced in [20]. In this report, the features, advantages, and disadvantages of each method are detailed.

Stereo vision is an effective method that is widely used in various fields. In [21], a linear method was developed for 3D line reconstruction using stereo vision. Reconstruction becomes difficult when the number of corresponding points on the lines is limited. To solve this challenge, the researchers in the study developed a method called point-then-direction (PtD). Their results show that the proposed method increased the accuracy of both the direction and the position of the 3D line reconstruction. In other research, a binocular stereo vision with Gaussian mixture model was introduced to recognize broccoli seeding [22]. In this study, some additional factors were considered during the procedure, such as weed conditions, shooting heights, and exposure intensities in the natural field. In addition to emphasizing the efficiency of the stereo vision method, the performance was reported as being satisfactory. Additionally, a famous method called structure from motion (SfM) was used for the 3D reconstruction of surfaces, in which the depth information within images was also involved [23,24].

With the advent of unmanned aerial vehicles, we are witnessing the use of these devices in the study of various phenomena in the agricultural fields, one of which is soil roughness. The data obtained from this tool have different resolutions that affect the roughness analysis, so more studies are needed to achieve more stable results.

In some methods, satellite and radar data are used to determine soil roughness. With careful processing of these data, acceptable results are usually obtained, but they are still considered expensive methods [25]. Additionally, these methods are still limited in their application in real time.

To overcome these challenges, a system based on the stereo vision technique was created to reconstruct the soil surface in the field in a 3D space in which the input images were common digital images in the visible band. Additionally, the use of conventional, rather than complex, methods to extract features and convert images to 3D mode was another objective of the present study.

## 2. Materials and Methods

### 2.1. Soil Preparation

In order to gather image data from tilled soil, farming operations were conducted in the field of Babolan (37°33′57″N 48°55′47″E at an altitude of 1352 m above sea level) located at 10 km from Ardabil city in 2018. The texture of the land and moisture content was sandy loam and 8%, respectively. To obtain a cloddiness structure for exploring roughness on the soil surface, the land was split into different plots. Each plot was then plowed by one of the primary and secondary tillage instruments: moldboard, rotavator, or disk plow. These machines provided different soil clods on the soil surface (fine to coarse grain size).

### 2.2. Image Acquisition

**Figure 1:**
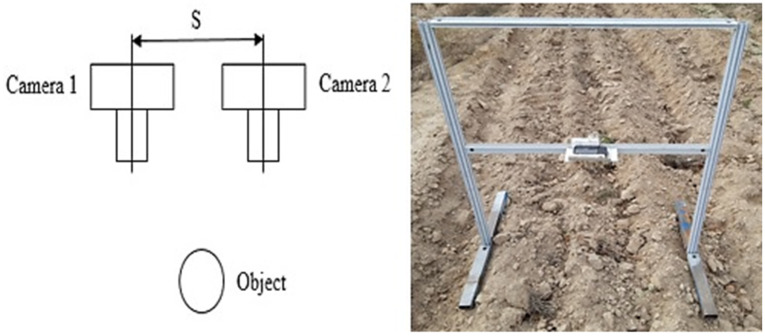
Schematic of the stereo imaging with its setup.

Since our goal was to gather the dataset in real field conditions, rather than in an isolated environment at laboratory, stereo-pair images were used. In this research, the W3-Fujifilm stereo camera, equipped with two 10-megapixel CCD sensors for both left and right lenses, was used with a baseline spacing of 7.5 cm, and a 12 mm focal length. The distance between the camera lens and the ground was 60 cm. The total number of stereo-pair images was 156, with a resolution of 3684 × 2736 pixels. A schematic image with a constructed platform for holding the camera is shown in Figure 1. Using this simple imaging device without the use of additional equipment, image data were collected, which was timesaving compared to similar methods such as DEM. Due to the use of three types of tillage tools (molboard, disk and rotavator), 52 images were prepared from each plot along with the measurement of the chain index.

### 2.3. Roughness Measurement

In order to avoid damaging the clods on the soil surface obtained from tillage operations, roughness was measured manually after imaging. For this, the roller chain method was used. The roller chain used in this study had joints of 4 millimeters with links of 5 millimeters and a total length of 1 m. This simple tool was carefully spread on the soil surface and the length covered after cultivation. The ratio between the distance over surface (1 m) and the Euclidean distance (measured by ruler) of the chain was used to calculate the chain roughness (CI index) as a measure of the roughness [25]. The CI index was calculated using the following equation:

(1) $$CI=\left(1-\frac{L_{1}}{L_{0}}\right)\times 100$$

where $L_{0}$ is the distance over surface (here 1 m) and $L_{1}$ is the Euclidean distance in meters [26]. It is worth noting that the CI index used in this study measured the height of plow layers to evaluate the result of the three-dimensional model. Indeed, the height of plow layers was calculated using both roller chain and 3D reconstruction.

### 2.4. Calibration and Image Rectification

The stereo algorithm was composed of four stages: calibration, rectification, corresponding, and triangulation. In order to increase the speed of all processing stages, the size of image data was reduced by four times. Stereo-pair images were calibrated by means of a chessboard pattern using the stereo camera calibrator toolbox of MATLAB R2018a and camera parameters were determined. The calibrated images were then rectified and both the left and right image were set in a horizontal line. This procedure minimized bias in extracted local features from stereo-pair images. More details are given in [27].

### 2.5. Keypoints Extraction and Matching

**Figure 2:**
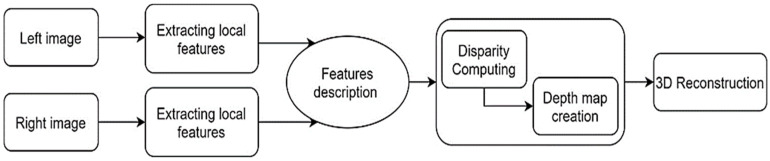
Pipeline of 3D reconstruction of tilled soil surface.

The pipeline of 3D reconstruction of soil surface after plowing operations is illustrated in Figure 2. In order to extract keypoints as effective features in stereo-pair images, the SIFT algorithm was applied. The main advantage of SIFT is that it is independent from rotation, scale, and robustness against affine transformation [28]. These keypoints reconstruct a 3D model named point cloud, whereby every point has three components along the x, y, and z axis. For implementing the SIFT algorithm, Python programming language and Numpy library were used.

In stereo systems, the coordinates of points are determined after matching the obtained keypoints from SIFT between left and right images, which are named corresponding keypoints. For this, the brute force algorithm was used so that the locations of all corresponding points were determined and a point cloud could be obtained. This algorithm computes Euclidean distance as matching criteria so that the minimum distance between points in the stereo-pair can be considered as a corresponding keypoint.

### 2.6. Disparity Calculation and Depth Map Estimation

**Figure 3:**
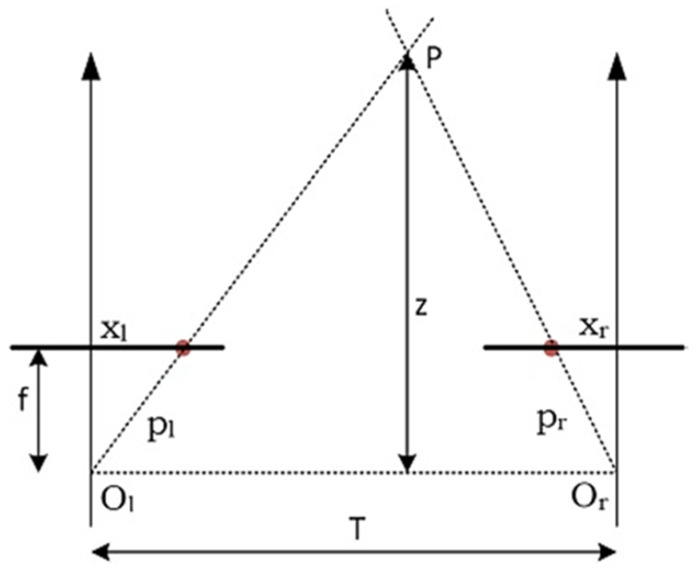
Graph of binocular disparity calculation from a single scene.

Disparity as a dissimilarity between the pixels of two left and right images was required to estimate depth because disparity is inverse to the distance between the observed objects inside images. Hence, disparity calculation is essential for the creation of depth map. For this, the binocular method was used where the disparity is the difference between the two images as shown in Figure 3.

(2) $$Disparity=\frac{fT}{z}=x_{l}-x_{r}$$

(3) $$z=\frac{fT}{\left|x_{l}-x_{r}\right|}$$

where f is the focal length of the camera, T is the distance between the origins of the two camera lenses, z is the depth value of the image, $X_{l}$ is the horizontal position of the pixel in the left image, and $X_{r}$ is the horizontal position of the matched pixel in the right image.

By calculating the depth of the images, the coordinates (*x*, *y*, *z*) of the objects are available and the scene can be reconstructed in 3D. Additionally, to determine pixel intensity as color, maximum depth was computed for all pixels and pixels with maximum depth were set to the value 0, represented by the color black. The color of each pixel was then calculated using the following equation:

(4) $$pixel\;color=255-\frac{255*depth}{maxdepth}$$

**Figure 4:**
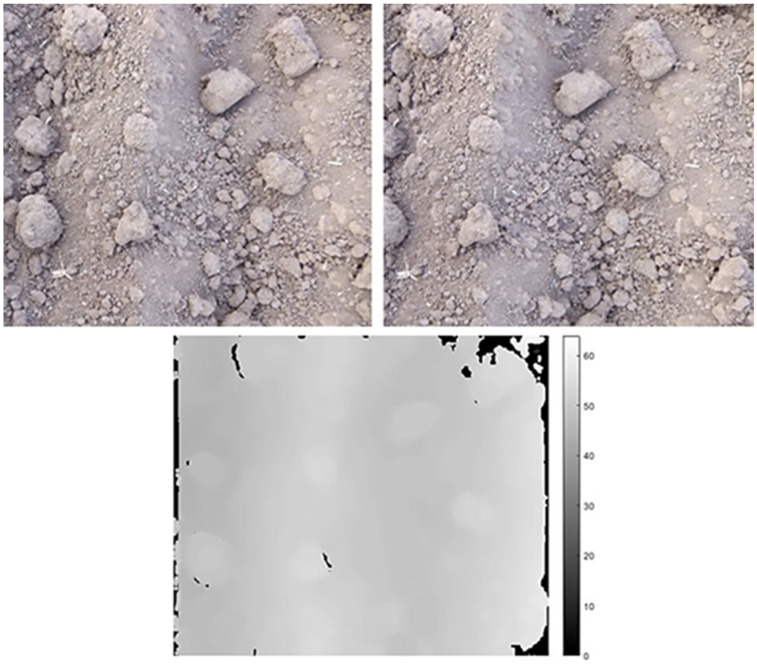
An example of stereo-pair image with corresponding depth image.

In the above formula, 255 was the value set to pixels in the left images which had no matched pixels in the right images. Thus, depth images were obtained so that in the images, darker regions represented objects which were far from the camera lens and the lighter regions represented the objects which were closer to the camera lens. A sample of depth maps is shown in Figure 4.

### 2.7. Three Dimmensional Reconstruction

The 3D reconstructed points from these images provide information about objects composed of continuous surfaces, from which the set of points are obtained [29]. To establish the relationship between these surfaces and the desired points, among various methods, triangulation was used as it is a simple and efficient method. For this, a linear relationship was set between the points of the image and the corresponding points of the flat surfaces, details of which are given in the study of [30]. The last step in 3D reconstruction was to apply textures to the surfaces formed in the triangulation step. The intensity for each point was determined based on Formula 4.

### 2.8. Evaluation Metric

As the output of our study has continuous rather than discrete values, regression was used to evaluate the results instead of classification. In other words, the values of plow layers obtained from CI and 3D model -which were continuous- were compared using a regression method to determine how much correlation there is between the operation and the constructed virtual 3D model. Therefore, a regression equation was used to evaluate the measured and estimated soil surface roughness. As the plowing layer heights were large enough to create considerable roughness, regression relationships were used to characterize the soil surface situation. Additionally, a determination coefficient was computed for each tillage implement. In order to quantitatively estimate the height of the plow layers, the peaks from the height profiles obtained from the depth map were detected and analyzed. If the heights of the peaks were at the same level, this was taken as an indication of plowing quality and roughness being in good condition. In addition, to validate the reconstructed 3D points obtained, the mean square error was calculated from the following equations. These relationships determine the difference between the values observed in the images ($x_{r}$) and the calculated values ($x_{cr}$).

(5) $$RMSE_{x}=\sqrt{\frac{\sum_{r=1}^{N}{\left(x_{cr}-x_{r}\right)}^{2}}{N}}$$

(6) $$RMSE_{y}=\sqrt{\frac{\sum_{r=1}^{N}{\left(y_{cr}-y_{r}\right)}^{2}}{N}}$$

(7) $$RMSE_{xy}=\sqrt{\overset{N}{\underset{r=1}{\sum }}\left({\left(RMSE_{x}\right)}^{2}+{\left(RMSE_{y}\right)}^{2}\right)}$$

## 3. Results

**Figure 5:**
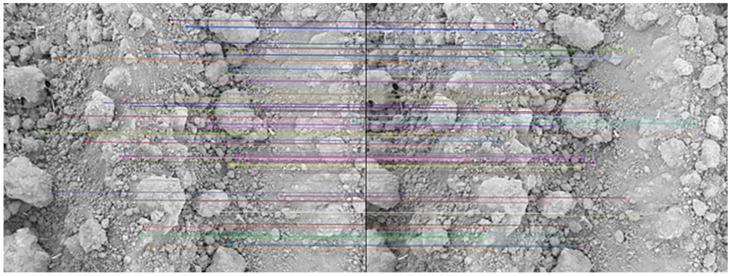
Illustration of matched keypoints using the brute force algorithm.

**Figure 6:**
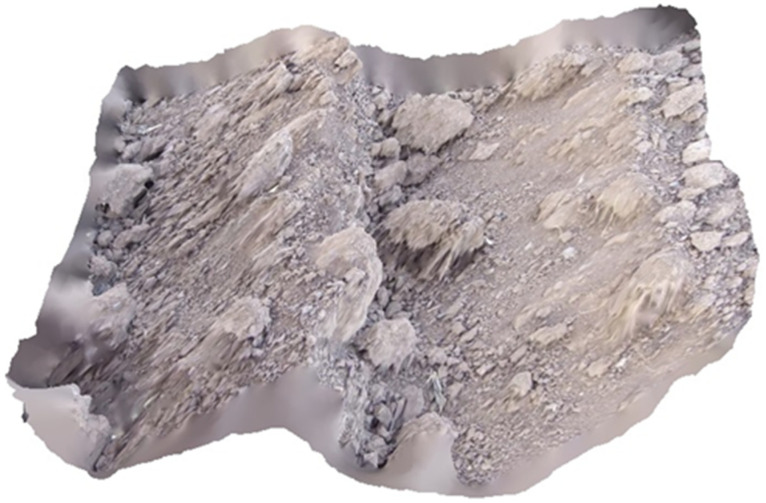
A sample of the 3D model obtained from the stereo image of soil surface of a tilled soil by the disk implement.

The visual results of extracted and matched keypoints are given in Figure 5. As can be seen, a sufficient number of keypoints were found. The 3D model of soil surface was reconstructed from the locations of the points and the differences on the horizontal lines between the left and right image. This is shown in Figure 6.

After constructing the three-dimensional model, we focused on evaluating the accuracy of the proposed method to determine its efficiency compared to actual measurement. For this purpose, and to evaluate the proposed method in the 3D reconstruction of soil surface images and validation of reconstructed 3D points, the root mean square error was used to calculate the accuracy of the set of points, by which the difference between the values (observed) in the images and calculated values are specified. Table 1 shows the values obtained from these relationships for 5 random points.

The results of evaluation are given in Table 2 containing regression equation with coefficients of determination. From the data in the table, it can be observed that the proposed method is an accurate method of estimating soil surface roughness in soil tilled by moldboards, disks, and rotavators. The R square values obtained were 0.90, 0.83, and 0.78 for moldboards, disks, and rotavators respectively. The reason for these values was that the size of soil aggregates and clods obtained from moldboard were larger than those obtained from the disk and rotavator. Thus, the elevation component of clods from the moldboard could be more accurately identified than the clods of the other instruments. In addition, the small size of soil aggregates obtained from rotavator and its high distance from camera lens led to the rotavator having the lowest R square value. This is consistent with the results of similar studies in this field [17].

Table 3 represents the results of calibration in the case of intrinsic and extrinsic parameters of the used stereo camera. The value of reprojection error was obtained as 0.61 and 0.59 for the first and second camera, respectively. Extrinsic parameters also included the rotation matrix and the transition vector, which were used to calculate the fundamental matrix and camera location, and finally, to calculate the three coordinates of the length, width, and height of the reconstructed 3D images. In this regard, the first camera (left) was considered as the base indicator and these vectors were calculated for the second camera (right).

**Figure 7:**
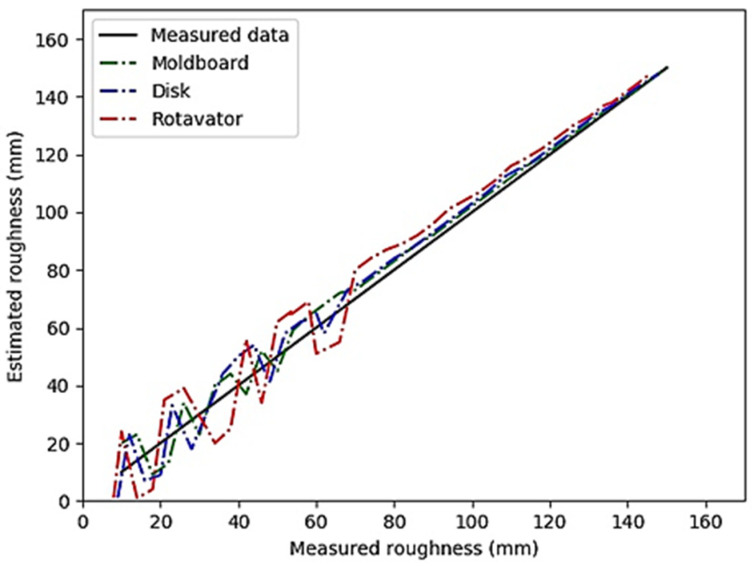
Comparison of the measured and estimated soil surface roughness for three tillage implements.

Figure 7 shows the regression graphs of three tillage implements in estimation of soil surface roughness. This graph indicates that the roughness obtained from moldboard was converged more effectively and more rapidly than the disk and rotavator because there was less distance between the soil surface and the camera lens for the moldboard than for the other two instruments. 

**Figure 8:**
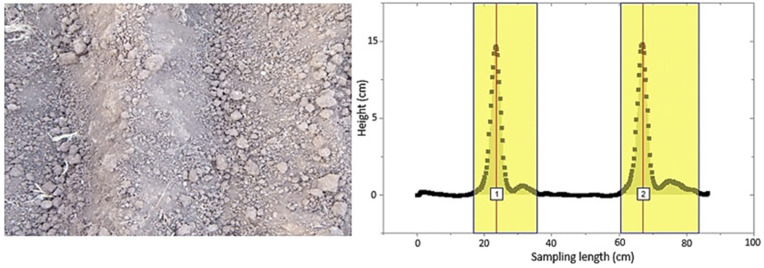
The original image of the surface of a plowed soil with the detection of peaks corresponding to the plow layers.

**Figure 9:**
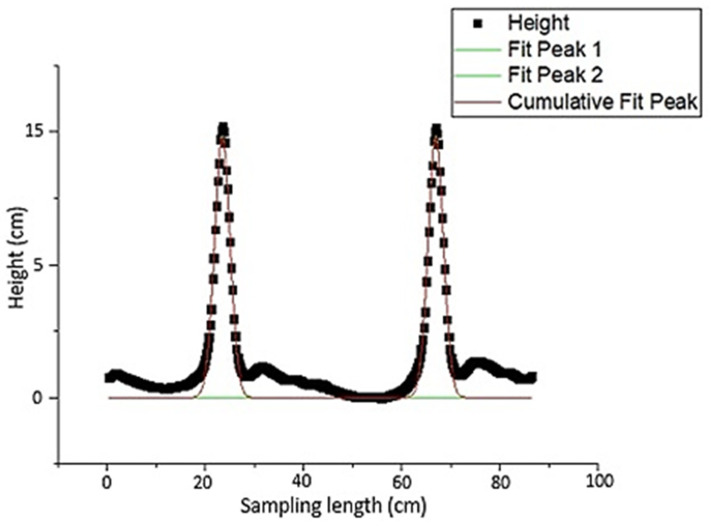
Corresponding peak fitting for two plow layers based on height profile.

**Figure 10:**
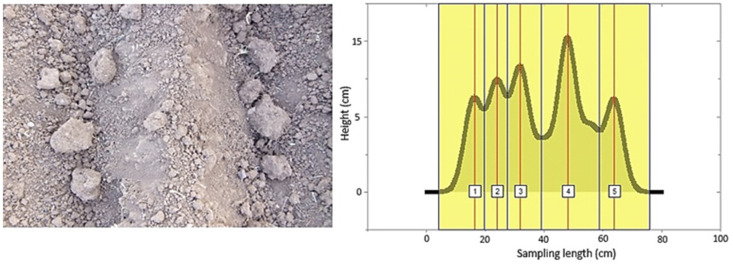
Irregular plow layers with the detection of peaks corresponding to the plow layers.

**Figure 11:**
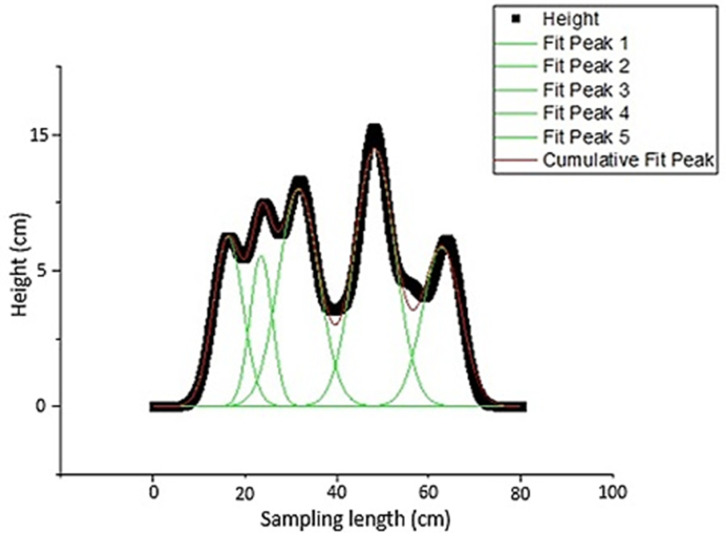
Corresponding peak fitting for the two irregular plow layers based on height profile.

Due to the previously given explanations, the height of plow layers, which is also a sign of soil surface roughness and plow quality, was examined. If the peaks were of the same height in the height profile, this was taken as an indication that the plowing quality and soil roughness were of good condition. For a plowed surface, the plow layers and corresponding peaks detected are shown in Figure 8. As seen in the figure, the two peaks were almost the same height. Therefore, it can be concluded that the crowns of the plow layers were at the same level. This indicates that the roughness and plow quality of the soil were in good condition. Figure 9 also shows a peak fitting. For profiles with relatively large differences in plow layer height, peak analysis and fitting are illustrated in Figure 10 and Figure 11. In these cases, the roughness and till quality was poor. It should be noted that the reason for the irregularities and differences between the predicted and measured values was due to the inaccuracy of the height component in the depth map, as well as the presence of noise that caused an unrealistic peak in the height profile. In other words, noise and inaccuracy in calculating the height component were the most significant variability factors in the peak analysis.

### 3.1. Noise Effect

It should be noted that in some cases, these irregularities and possibly peaks are due to noise in the images. In these cases, if the amount of noise is above a certain level, a pre-processing step, such as noise removal, should be applied to the images. In this study, the level and type of noise is not known and needs further investigation.

Proper selection of tillage machine and proper implementation of plowing operations will lead us to a desirable soil roughness that can be confirmed by accurate imaging without noise and side effects and accurate processing and analysis.

### 3.2. Robustness against Geometrical Variations

The SIFT algorithm was used to extract local features to make three-dimensional soil surface images robust against different geometric variations, such as translation, rotation, scale, and affine transformation. These advantages facilitated the work during data collection. However, other feature detectors, such as SURF, should be implemented to improve their performance.

## 4. Discussion

Ideally, the average squares of the error of the image points should be less than the size of a pixel, i.e., subpixels, but in practice such a degree of accuracy does not always occur due to reasons such as model defects. Additionally, a large error value for a particular point indicates the incorrect path of the algorithm for that point in terms of incorrect coordinates of the corresponding points or the placement of points in the wrong place. It should be noted, however, that the RMSE is not a systematic error, and its value cannot be predicted in advance or reduced by standardization processes, such as calibration. Therefore, it cannot be concluded that a point with a high RMSE is necessarily the least accurate point among a set of points.

From the stereo vision perspective, due to the small amount of disparity (difference between the horizontal distance of the corresponding points in the two images left and right) between the plow layers of the disc and the rotavator, the depth is greater. As a result, in the stereo vision system, layers that were further away from the camera lens had insufficient depth and resolution accuracy. For a similar reason, the depth map for the plow layers of the moldboard had higher resolution. This is also consistent with the human visual perception of the environment and the rule that larger objects are better seen and analyzed. Additionally, the correlation coefficients of the three tillage machines were obtained as 0.95, 0.91 and 0.89 respectively, showing a strong correlation between the actual and estimated roughness values. The regression results show an improvement compared to the similar study in which regression equations were used to characterize clods and soil aggregates [11].

Regarding camera calibration, the amount of reprojection error was acceptable for estimating the stereo camera parameters, and was suitable for 3D image reconstruction, which was also consistent with the reports of [31,32]. This means that there was no need for recalibrating.

The required time for 3D reconstruction was about 2500 ms for a pair of stereo images. It is worth nothing that the time can be changed due to factors such as point cloud volume, the number of meshes of the triangulation, the used hardware, etc., so that if the processing power was high, the time required to obtain the result would be reduced.

In the peak analysis, it should be noted that the reason for the irregularities and differences between the predicted and measured values is due to the inaccuracy of the height component in the depth map, as well as the presence of noise that caused an unrealistic peak in the height profile. The source and amount of noise is not known, but its existence does not need to be proven, especially since the images were taken in ambient conditions.

## 5. Conclusions

In this study, soil surface roughness, including random and oriented roughness, was obtained from three tillage instruments as a combination of primary and secondary tillage was estimated. To achieve this aim, a new dataset from the real conditions of the field was gathered, and a stereo vision approach was applied to the dataset as a non-contact method. The elevation component was computed, and the dataset was reconstructed as a 3D model. Following this, keypoints as local features were extracted from the images using SIFT technique. By matching and refining corresponding features, depth math containing the elevation component was computed. Finally, 3D reconstruction of the tilled soil was obtained using triangulation. It can be concluded that estimation of soil surface roughness via RGB images is not only feasible but also satisfactory if it is properly calibrated. Another finding of this study was that the height of the plow layers could be a good indicator of the ruggedness and quality of tilled soil. One of the limitations of this study was poor hardware, which had a negative effect on the duration of the 3D map creation. Adjustment and obtaining the optimal soil roughness, which depends on many factors such as plant type and physical parameters of the soil, should be investigated in future research.

## Figures and Tables

**Table 1:** RMSE values for 5 random points from the plowed soil surface in the 3D reconstructed image.

Random Points of Soil Surface	RMSE *X* (Pixel)		RMSE *Y* (Pixel)	
	*X*1	*X*2	*Y*1	*Y*2
1	2.12	2.09	0.91	0.86
2	2.36	2.42	1.22	1.40
3	0.98	0.85	1.75	2.08
4	1.79	1.93	0.68	0.80
5	1.90	2.23	0.74	1.01
Mean RMSE	1.07		1.65	
RMSE *XY*	2.14			

**Table 2:** Values of regression equation of the proposed method in estimation of soil surface roughness.

Evaluation Metrics	Implement type		
	Moldboard	Disk	Rotavator
R^2^	0.9	0.83	0.78
RMSE	9.08	10.83	12.32

**Table 3:** Calibration results and estimation of intrinsic and extrinsic parameters of the stereo camera.

Parameters	Intrinsic Parameters		Extrinsic Parameters	
	Camera 1	Camera 2	Rotation Vector (°)	Translation Vector (mm)
Focal length (pixels)	(61.4, 61.4)	(61.4, 61.4)	(0.003, 0.002, 1.670 × 10^−4^)	(−73.080, 1.009, −9.143)
Principle point (pixels)	(54.6, 75.6)	(54.7, 74.8)		
Skew value (pixels)	(0.9078)	(0.9016)		
Radial distortion (mm)	(0.0167, 0.4256)	(0.0167, 0.4282)		
Tangential distortion (mm)	(6.84 × 10^−4^, 5.× 10^−4^)	(6.83 × 10^−4^, 5.81 × 10^−4^)		

## Data Availability

The data presented in this study are available on request from the corresponding author.
